# Evaluation of a method to identify midwives in national provider identifier data

**DOI:** 10.1186/s12884-023-06122-2

**Published:** 2023-11-22

**Authors:** Jennifer Vanderlaan, Karen Jefferson

**Affiliations:** 1 University of Nevada Las Vegas School of Nursing, 4505 S. Maryland Parkway, Box 453018, Las Vegas, NV 89154-3018 USA; 2 American College of Nurse-Midwives, 409 12Th St SW, Suite 600, Washington, DC 20024-2188 USA

**Keywords:** Midwifery, Health Workforce, Nurses, Information Sources

## Abstract

**Objectives:**

Comparison of national midwife workforce data from the National Provider Identifier file determined it undercounted midwives compared to national data available from the American Midwifery Certification Board. This undercount may be due to the existence of three taxonomy categories for midwives when registering for the National Provider Identifier. The objective of this study was to obtain an accurate count of advanced practice midwives using the National Provider Identifier Data.

**Methods:**

A recode strategy was created using the NPPES Data Dissemination File for November 7, 2021. The strategy identified advanced practice midwives using education and certification information provided in the “credentials” field. The strategy was validated using the NPPES Data Dissemination File for August 7, 2022 and the gold standard was the American Midwifery Certification Board count of midwives by state for August, 2022. Validation compared the accuracy and precision of the recode to the accuracy and precision of using the advanced practice midwife taxonomy category.

**Results:**

The recode strategy improved the accuracy and precision of the count of advanced practice midwives compared to the identification of advanced practice midwives using the advanced practice midwife taxonomy category.

**Conclusions for practice:**

Recoding the NPPES Data Dissemination File provides a more accurate and precise count of advanced practice midwives than relying on the existing advanced practice midwife taxonomy classification. Researchers can use the NPPES Data Dissemination File when studying the midwifery workforce.

**Supplementary Information:**

The online version contains supplementary material available at 10.1186/s12884-023-06122-2.

## Significance

*What is already known on this subject?*

The National Provider Identifier file is a national list of healthcare providers with practice location. The National Provider Identifier file contains only 60% of the advanced practice midwives (certified nurse-midwives and certified midwives) certified with the American Midwifery Certification Board, reducing its usefulness for research and planning.

*What this study adds?*

Additional advanced practice midwives were identified in the National Provider Identifier File under a different midwife taxonomy code.  Recoding midwives using both the taxonomy codes and the credential text improves accuracy of the NPI data for research and planning.

**Supplementary Information:**

The online version contains supplementary material available at 10.1186/s12884-023-06122-2.

## Introduction

Quantifying the size and distribution of the healthcare workforce is vital to projecting shortages and filling needed gaps in care, yet workforce reporting isn’t always accurate [1]. In a review of national data on the midwifery workforce, Ranchoff and Declercq reported that the midwifery workforce data provided in the Area Health Resource File (AHRF) was undercounting midwives in each state [2]. There was no discernable pattern in the undercount with the AHRF. In total, the AHRF reported only 60% of the number of midwives included in the counts of midwives provided by the American Midwifery Certification Board (AMCB), the national organization responsible for certifying nurse-midwives and certified midwives.

The AHRF is a tool provided by the Bureau of Health Workforce at the U.S. Health Resources and Services Administration. The AHRF aggregates health services data from over 50 sources to provide county and state level information [3]. Each years’ county files include the number of health care providers, health facilities, population characteristics, hospital utilization, hospital expenditures, and environment characteristics by county in the United States. The AHRF is used by health planners and health advocacy organizations. Data included in the AHRF are used in the identification of Health Provider Shortage Areas, also referred to as HPSAs. Health Provider Shortage Area designation is used to determine eligibility for some federal funding, for example student loan repayment programs for nurses. Health Provider Shortage Area designation is also used to determine eligibility for some Centers for Medicare and Medicaid Services reimbursement programs [4]. The March of Dimes uses the AHRF to measure maternity care deserts, an advocacy tool for raising public and policy-maker interest in addressing the national shortage of maternity care [5].

Researchers examining system-level associations with the health workforce and maternal outcomes, such as maternal mortality, use the AHRF because it provides a single source of health provider, facility, and community data [6]. The AHRF is a free source of county-level health provider data, and the only source of county-level midwife data. Researchers rely on the AHRF to identify associations between state regulation of midwives and the size and distribution of the midwifery workforce [7, 8]. These data are also necessary to examine associations between variations in midwifery regulation and pregnancy outcomes because these associations are modified by the density of the midwifery workforce [9]. The undercount of midwives in the AHRF increases risk of misclassification bias in midwifery workforce and policy research. Nondifferential misclassification, that is misclassification with no clear pattern, usually reduces the observed difference between the experimental and control group causing misclassification bias [10]. Understanding the cause of the undercount, and how to correct it, may improve the accuracy of evidence produced by health policy researchers and improve reporting of the distribution of maternal health resources.

When considering the reasons for the undercount in the AHRF relative to the AMCB data, one possible reason could be inaccuracy of the AMCB certification data. About 20% of midwives certified with the AMCB are not currently employed in the discipline of midwifery or are unemployed in the year before recertification [11]. While this may account for a 20% undercount in the AHRF, it should not result in the undercount reported by Ranchoff and Declerq [2]. Midwives recertify with AMCB every five years, which may result in data lag if midwives leave clinical practice or move to another state. If data lag were the cause of the discrepancy, the AHRF should both overcount and undercount midwives. Ranchoff and Declerq only found undercounting, which means data lag is unlikely to be the source of the discrepancy.

The other possible source of the undercount in the AHRF relative to the AMCB data is inaccuracy in the data source for the AHRF. The data source for midwives in the Area Health Resources File is the National Provider Identifier (NPI) Registry. The NPI number serves as a unique identifier for each health provider for use in electronic health communication, such as with billing third-party insurers [12]. Midwives applying for an NPI number select between three midwife taxonomy codes that distinguish midwives based on certification [13]. Midwives certified with the AMCB register as “advanced practice midwives”, which is a subcategory of physician’s assistants and advanced practice nursing providers. The other two midwifery taxonomy codes, “midwife” and “lay midwife,” have general definitions that do not indicate specific licensure or certification. Because the regulations and scope of practice are different for midwives with different certifications, the three categories should result in a data source that provides information about both the size and practice scope of the midwifery workforce. The AHRF counts midwives by identifying the number of NPIs with the taxonomy code for advanced practice midwives [3]. If midwives certified through AMCB are registering for NPIs using one of the other two midwife taxonomy categories, this registration error could be responsible for the undercount of midwives.

The objective of this study was to increase the accuracy of counting advanced practice midwives using the National Provider Index data. If a more accurate count can be obtained, the recoded data would better reflect the distribution of advanced practice midwives throughout the country and prevent research bias from misclassification of the count of midwives in each state.

## Methods

This was a cross-sectional study of advanced practice midwives in the United States.

### Procedure for writing the recode

National Provider Identifier (NPI) data for this study was obtained from the Centers for Medicare and Medicaid publicly available National Plan and Provider Enumeration System (NPPES) Data Dissemination file [14]. The NPPES Data Dissemination file includes the provider taxonomy code and a field to enter provider credentials. There is also a field for the provider to indicate the state of license. The public file is updated monthly and made available free for download. The recode strategy was written using the November 7, 2021 file.

The procedure for designing the recode of advanced practice midwives was as follows:

1. Create frequency tables of the credentials reported by all providers as Advanced Practice Midwife (367A00000X), Midwife (176B00000X), or Lay Midwife (175M00000X).
2. Sort the text in the credentials field based on license, certification, or education terms. Text that indicated licensing (e.g. APRN), education (e.g. MSN), or credentials (e.g. CNM) as a certified nurse-midwife or certified midwife was grouped as identifying advanced practice midwives. Text that indicated education or training as a nurse was interpreted as likely to mean credentials of certified nurse-midwife and were recoded as advanced practice midwife. Credentials that indicated licensing or certification as a certified professional midwife and those whose scope of practice is similar such as direct entry midwife or lay midwife, were recoded as other midwives.
3. Identify state-specific midwifery registration and titles that may result in misclassification. For example, certified professional midwives in New Hampshire are licensed using the title certified midwife. A state-specific recode differentiated advanced practice midwives and other midwives when necessary.
4. Identify credentials of a nurse-midwife for providers registered within the nurse practitioner taxonomy. A nurse-practitioner specific recode identified misclassified advanced practice midwives.

A program, written for SAS v 9.4, categorized midwives as advanced practice midwives if 1) there was any credential text that confirmed advanced practice midwife credentials, or 2) the midwife registered as an advanced practice midwife but did not include text information for credentials. The program categorized nurse practitioners as advanced practice midwives only if the credential indicated nurse-midwife. The program used for the recode is available in the supplement.

### Validation of recode strategy

Data to validate the recode strategy was abstracted from the American Midwifery Certification Board (AMCB) Research Report for August 2022 [15]. The AMCB is the certification body for certified midwives and certified nurse-midwives. Midwives recertify with the AMCB every five years. The number of currently certified midwives is reported publicly three times per year on the website. Midwives were eligible to be included if they were counted in one of the 50 U.S. states or the District of Columbia. Midwives who listed their state as a branch of the military, one of the other U.S. Territories, or “international” were excluded from the analysis.

A validation set for the NPI recode was created by applying the recode strategy to the Full Replacement Monthly NPI File for August 7, 2022. Validation included evaluation of the accuracy and precision of the recode compared to the original taxonomy coding. Accuracy was measured as the average error of the count of midwives from the AMCB count of midwives by state, which is the mean difference. Precision was measured as the average deviation of the count of midwives from the AMCB count of midwives by state. The recode would be considered valid if the recode resulted in a statistically significant improvement in the accuracy and precision, measured by a paired samples T-test.

Finally, the magnitude of the change was reported as the change in midwife density in each state. Density of midwives was measured as the number of midwives per 1,000 live births for the year 2020, the most recent year of data available for public use at the time of analysis [16]. The magnitude of the change was measured using a paired samples T-test, with a statistically significant reduction in the mean difference from the AMCB density of midwives interpreted as an improvement.

## Results

The AMCB reported 13,888 active midwives in August, 2022. Of these, 13,791 were located in one of the 50 U.S. States or the District of Columbia. Using the original taxonomy codes, the August 2022 NPI Data file included records for 9,933 advanced practice midwives, 5,658 midwives, and 413 lay midwives in U.S. States and the District of Columbia.

The NPI recode identified 12,117 midwives as advanced practice midwives. Of these, 10,711 (88.4%) were able to be verified with text in the credential field. The midwives verified as advanced practice included 8,729 (81.5%) registered using the advanced practice midwife taxonomy, 1,963 (18.3%) registered using the midwife taxonomy, and 19 (0.2%) registered using the lay midwife taxonomy. A total of 1,182 (11.9%) midwives registered with the advanced practice taxonomy did not have text that could be used to verify credentials. These midwives remained coded as advanced practice midwives because the taxonomy code description includes AMCB certification so it was unlikely midwives with other certifications would register for this category [13]. Midwives registered with the midwife or lay midwife taxonomy whose credentials could not be verified were recoded as other midwife. Additionally, 224 NPI registrants with credentials that match advanced practice midwives were found registered using the taxonomy code for nurse practitioner. Results of the recode can be found in Table 1.

**Table 1:** Distribution of midwives by NPI taxonomy category according to credentials provided in the NPPES Data Dissemination file, August, 2022

	**Taxonomy Category**		
**Recode Verification**	**Advanced Practice Midwife** ***n* = 9,933**	**Midwife** ***n* = 5,658**	**Lay Midwife** ***n* = 413**
Advanced Practice Midwife	8,729 (88.0%)	1,963 (34.7%)	19 (4.6%)
Other Midwife	22 (0.2%)	2,380 (42.1%)	217 (52.5%)
Unable to Verify	1,182 (11.9%)	1,315 (23.2%)	177 (42.9%)

Advanced practice midwives are certified as certified nurse-midwives or certified midwives with the American Midwifery Certification Board (AMCB)

### Validation results

The NPI recode changed the count of midwives in every state. The smallest improvement was 2 additional midwives (South Carolina and Wyoming). The largest change was the addition of 364 midwives (New York). Compared to the AMCB count of midwives, the original NPI taxonomy coding resulted in undercount of 100 or more midwives in thirteen states and an overcount in two. The recode resulted in undercount of 100 or more midwives in four states and an overcount in seven. Full results of the recode by state are reported in Table 2.

**Table 2:** Comparison of accuracy of the count of advanced practice midwives in the NPI file by taxonomy categorization and after applying a recode^a^

State	AMCB Count^b^	Taxonomy Count^c^	Taxonomy Absolute Difference	Taxonomy Relative Difference	Recode Count^d^	Recode Absolute Difference	Recode Relative Difference
Alabama	61	29	-32	-52.5%	38	-23	-37.7%
Alaska	116	76	-40	-34.5%	90	-26	-22.4%
Arizona	285	203	-82	-28.8%	226	-59	-20.7%
Arkansas	45	29	-16	-35.6%	36	-9	-20.0%
California	1235	777	-458	-37.1%	968	-267	-21.6%
Colorado	486	350	-136	-28.0%	417	-69	-14.2%
Connecticut	225	190	-35	-15.6%	218	-7	-3.1%
Delaware	46	31	-15	-32.6%	38	-8	-17.4%
District of Columbia	55	65	10	18.2%	56	1	1.8%
Florida	839	603	-236	-28.1%	696	-143	-17.0%
Georgia	597	433	-164	-27.5%	529	-68	-11.4%
Hawaii	57	39	-18	-31.6%	46	-11	-19.3%
Idaho	84	56	-28	-33.3%	74	-10	-11.9%
Illinois	502	404	-98	-19.5%	461	-41	-8.2%
Indiana	213	187	-26	-12.2%	202	-11	-5.2%
Iowa	133	112	-21	-15.8%	126	-7	-5.3%
Kansas	93	60	-33	-35.5%	67	-26	-28.0%
Kentucky	134	104	-30	-22.4%	108	-26	-19.4%
Louisiana	74	38	-36	-48.6%	47	-27	-36.5%
Maine	113	87	-26	-23.0%	126	13	11.5%
Maryland	336	259	-77	-22.9%	319	-17	-5.1%
Massachusetts	453	376	-77	-17.0%	486	33	7.3%
Michigan	490	359	-131	-26.7%	428	-62	-12.7%
Minnesota	399	296	-103	-25.8%	350	-49	-12.3%
Mississippi	32	28	-4	-12.5%	43	11	34.4%
Missouri	132	87	-45	-34.1%	109	-23	-17.4%
Montana	56	52	-4	-7.1%	65	9	16.1%
Nebraska	57	40	-17	-29.8%	51	-6	-10.5%
Nevada	59	46	-13	-22.0%	58	-1	-1.7%
New Hampshire	117	107	-10	-8.5%	110	-7	-6.0%
New Jersey	326	233	-93	-28.5%	292	-34	-10.4%
New Mexico	210	168	-42	-20.0%	232	22	10.5%
New York	1060	657	-403	-38.0%	1021	-39	-3.7%
North Carolina	513	388	-125	-24.4%	458	-55	-10.7%
North Dakota	22	26	4	18.2%	22	0	0.0%
Ohio	480	334	-146	-30.4%	416	-64	-13.3%
Oklahoma	78	56	-22	-28.2%	65	-13	-16.7%
Oregon	379	299	-80	-21.1%	351	-28	-7.4%
Pennsylvania	550	370	-180	-32.7%	500	-50	-9.1%
Rhode Island	83	58	-25	-30.1%	72	-11	-13.3%
South Carolina	141	100	-41	-29.1%	102	-39	-27.7%
South Dakota	42	31	-11	-26.2%	46	4	9.5%
Tennessee	305	207	-98	-32.1%	233	-72	-23.6%
Texas	582	477	-105	-18.0%	538	-44	-7.6%
Utah	186	129	-57	-30.6%	169	-17	-9.1%
Vermont	95	65	-30	-31.6%	78	-17	-17.9%
Virginia	373	230	-143	-38.3%	268	-105	-28.2%
Washington	482	331	-151	-31.3%	358	-124	-25.7%
West Virginia	72	44	-28	-38.9%	63	-9	-12.5%
Wisconsin	262	189	-73	-27.9%	224	-38	-14.5%
Wyoming	26	18	-8	-30.8%	20	-6	-23.1%

^a^Data Source NPEES Data Dissemination Full Replacement Monthly NPI File for August 7, 2022

^b^AMCB – The American Midwifery Certification Board certifies advanced practice midwives (certified nurse-midwives and certified midwives). Data from the August 2022 report of count of midwives

^c^Taxonomy refers to midwives registered for NPI numbers using taxonomy code 367A00000X, Advanced Practice Midwife

^d^Recode refers to midwives identified as advanced practice midwives using taxonomy code and credentials text

The NPI recode improved the NPI accuracy and precision relative to the AMCB reported number of midwives. The mean difference for the original NPI taxonomy code was 75.6 (SD 91.0). The absolute error ranged from an overcount of 10 midwives to an undercount of 458 midwives while the relative error ranged from an 18.2% overcount to a 52.5% undercount. The mean difference for the NPI recode was 32.8 (SD 47.9). The absolute error ranged from an overcount of 33 midwives to an undercount of 267 midwives while the relative error ranged from 34.4% overcount in Mississippi to 37.7% undercount in Alabama. The paired samples T-test calculated there was a statistically significant change in the mean difference, which was interpreted as an improvement (Mean Change -42.8; 95% CI –26.0 – -59.6; *p* < 0.001).

The first step to calculate the magnitude of the change (i.e. the change in mean difference in density), was to calculate the mean difference in density between the AMCB report and both the original NPI and the NPI recode. The AMCB report of midwives presented a mean density of 4.8 midwives per 1,000 births with a range from 0.9 midwives to 18.5 midwives. The original NPI resulted in a mean density of midwives at 3.6 per 1,000 births with a range from 0.5 to 12.7. The mean difference between the AMCB density and the original NPI density was 1.19 (SD 1.03; *p* < 0.001), which was a statistically significant difference. The NPI recode resulted in a mean density of midwives at 4.3 per 1,000 births with a range from 0.7 to 15.2. The mean difference between the AMCB density and the NPI recode was 0.48 (SD 0.73; *p* < 0.001). The magnitude of the change was then calculated by comparing the two measurements using a mean difference. The NPI recode was 0.7 midwives per 1,000 births (95% CI -0.9 – -0.5; *p* < 0.001) more accurate than the original NPI coding. The result of calculations for density by state can be seen in Table 3.

**Table 3:** Density of advanced practice midwives per 1,000 live births per state as measured by AMCB certification records August 2022, NPI August 2022 Datafile original taxonomy and NPI Datafile recode^a^

State	AMCB Midwives^b^	NPI Original Taxonomy^c^	NPI Recode^d^
Alabama	1.06	0.50	0.66
Alaska	12.25	8.03	9.50
Arizona	3.70	2.64	2.94
Arkansas	1.28	0.82	1.02
California	2.94	1.85	2.30
Colorado	7.90	5.69	6.78
Connecticut	6.72	5.68	6.52
Delaware	4.43	2.98	3.66
District of Columbia	6.20	7.32	6.31
Florida	4.00	2.88	3.32
Georgia	4.87	3.54	4.32
Hawaii	3.61	2.47	2.91
Idaho	3.90	2.60	3.44
Illinois	3.77	3.03	3.46
Indiana	2.71	2.38	2.57
Iowa	3.68	3.10	3.49
Kansas	2.71	1.75	1.95
Kentucky	2.59	2.01	2.09
Louisiana	1.29	0.66	0.82
Maine	9.79	7.54	10.92
Maryland	4.90	3.78	4.65
Massachusetts	6.82	5.66	7.32
Michigan	4.71	3.45	4.11
Minnesota	6.29	4.67	5.52
Mississippi	0.90	0.79	1.21
Missouri	1.91	1.26	1.57
Montana	5.19	4.82	6.02
Nebraska	2.35	1.65	2.10
Nevada	1.75	1.37	1.72
New Hampshire	9.92	9.07	9.33
New Jersey	3.33	2.38	2.98
New Mexico	9.59	7.67	10.59
New York	5.06	3.14	4.88
North Carolina	4.39	3.32	3.92
North Dakota	2.19	2.58	2.19
Ohio	3.72	2.59	3.22
Oklahoma	1.64	1.18	1.36
Oregon	9.52	7.51	8.81
Pennsylvania	4.21	2.83	3.83
Rhode Island	8.22	5.74	7.13
South Carolina	2.53	1.80	1.83
South Dakota	3.83	2.83	4.20
Tennessee	3.88	2.63	2.96
Texas	1.58	1.30	1.46
Utah	4.07	2.82	3.70
Vermont	18.51	12.66	15.20
Virginia	3.94	2.43	2.83
Washington	5.80	3.98	4.31
West Virginia	4.16	2.54	3.64
Wisconsin	4.32	3.12	3.70
Wyoming	4.24	2.94	3.26

^a^Density of advanced practice midwives per state calculated with 2020 birth certificate records counts of live births

^b^AMCB – The American Midwifery Certification Board certifies advanced practice midwives (certified nurse-midwives and certified midwives). Data from the August 2022 report of count of midwives

^c^Data Source NPEES Data Dissemination Full Replacement Monthly NPI File for August 7, 2022. Taxonomy refers to midwives registered for NPI numbers using taxonomy code 367A00000X, Advanced Practice Midwife

^d^Recode refers to midwives identified as advanced practice midwives using taxonomy code and credentials text

## Discussion

This study demonstrated that it is feasible to recode the National Provider Identifier (NPI) publicly available data file to create a database of the midwifery workforce that more closely agrees with the workforce numbers provided by AMCB. The method of recoding based on credential text included in the datafile identified 2,151 certified nurse-midwives and 58 certified midwives whose credentials indicate their registration would more accurately fall under the taxonomy code for advanced practice midwife. This technique can be used to obtain more accurate counts of midwives in the United States.

The NPI recode improved the agreement of the number of midwives in the NPI file with the number of midwives reported by AMCB. It is unlikely the two records of the number of midwives will be the same. This is primarily because the AMCB data includes midwives who are not currently working as midwives and midwives who are working in non-clinical midwife jobs such as administration, research, or education. These midwives may not maintain an NPI, which is primarily used for billing of clinical services. The timeframes for the two data are also different. AMCB data includes information from five years of certifications. Midwives who make changes in location or work during the five-year certification window may not update this information until their next recertification.

The main benefit of using NPI data to study the midwifery workforce is the availability of obtaining the number of midwives within each county. The United States government uses information about the local size and distribution of the health workforce to identify areas in need of federal funding to increase access to health care services. According to the World Health Organization, indicators used to track the health workforce should be SMART: Specific, measurable, attainable, relevant, and timebound [1]. The public monthly updated NPI file achieves the measurable, attainable, relevant, and timebound requirements. Recoding the NPI datafile to identify advanced practice midwives improves the specificity of the count of advanced practice midwives. The NPI datafile includes a field for the zip code, allowing precise measures of the midwifery workforce in smaller geographic units than is available publicly from the American Midwifery Certification Board.

Another benefit to using the NPI data to study the midwifery workforce is the inclusion of all midwives. Like physicians, midwives in the United States have two educational pathways that use different examinations to demonstrate proficiency. The AHRF only includes midwives trained in programs accredited by the Accreditation Commission for Midwifery Education (ACME) and certified by the American Midwifery Certification Board (AMCB). These midwives provide care for 10.6% of births in the United States, and 95% of the births attended by these midwives are in hospitals [16]. Midwives may also enter the profession through certification by the North American Registry of Midwives (NARM) after education through a portfolio evaluation process or a Midwifery Education Accreditation Council (MEAC) accredited program. These midwives provide care for 1.2% of births in the United States, all in homes or birth centers [16]. Because these midwives are not included in the AHRF, they are not included in the assessment of maternity care deserts or Health Provider Shortage Areas, which can affect federal funding decisions for midwifery education and clinical services.

In this study, 20% of midwives certified by the AMCB had registered for an NPI number in the wrong midwife category. This may be because the term advanced practice midwife is only used in the NPI taxonomy categories; it is not used by any credentialing body or state regulating body. In contrast, some titles for midwives have multiple uses. For example, the AMCB uses the term Certified Midwife for master’s prepared midwives without a prior degree in nursing. The state of New Hampshire uses the term Certified Midwife for midwives certified by the North American Registry of Midwives [17]. Though both midwives are identified as certified midwife in New Hampshire only midwives with AMCB certification practice full scope midwifery care throughout the life span and are clinically educated to attend births in hospitals. The lack of standardization of midwifery titles across states may contribute to inaccurate measurement of the size and distribution of the workforce.

Another challenge to accurately measuring the size and distribution of the midwifery workforce is the variation created by state differences in midwifery licensure, prescribing, scope of practice, and reimbursement. Though certified nurse-midwives can be licensed in any state, certified midwives can currently be licensed for practice in eleven states and the District of Columbia. Some states like New Jersey and Ohio require a physician colleague to review a midwife’s patient charts [18, 19]. Missouri requires a physician to establish a plan of care before a patient can begin care with a midwife [20]. Missouri, and Texas require midwives to prescribe as an act delegated by a physician rather than providing independent prescriptive authority for midwives [20, 21]. In twenty-one states, care provided by midwives is reimbursed by Medicaid at 75–95% the rate reimbursed to physicians for the same care [22]. Some states require that hospital admission be completed by a physician, even if the patient is under midwifery care. Each of these rules provides an incentive for midwives and their physician colleagues to attribute the work of midwives to physicians. These regulations may be contributing to the inaccuracy in the NPI measurement of the size and distribution of the midwifery workforce if midwives do not obtain and use their own NPI number. This may explain some of the residual state variation in absolute and relative difference after the NPI recode. Similarly, some residual variation may be caused by differences in non-clinical employment opportunities for midwives. The most recent AMCB report on midwifery employment indicates 22.7% of midwives work in jobs with no clinical responsibility [23].

This study highlights opportunities for meaningful use of NPI data. The current study demonstrated that the NPI file can be considered a census of midwives in the United States, supporting the validity of prior studies that describe the healthcare workforce with NPI data [24, 25]. This finding supports recommendations that NPI numbers be expanded to include nurses without advanced practice status as a way to better understand the health system [26]. Similarly, providing NPI taxonomy categories for members of the health workforce who fill research, administration, and education roles, not just clinical roles, can allow better assessment of the size of and scope of the health workforce [26]. For example, one of the potential reasons for disagreement between the number of midwives certified and the number of midwives with NPIs in the current study is that midwives without clinical responsibilities do not need an NPI.

The current study also supports the growing body of evidence of inaccuracies in NPI provider categorizations [27, 28]. These problems are similar to barriers to meaningful use reported for electronic health record data – missing data and lack of standards – and can be addressed through quality improvement initiatives [29]. In the case of midwifery, the multiple pathways to the profession and lack of standardization of midwife licensing will likely hinder NPI data quality improvement. Multiple pathways to midwifery results in confusion even among midwives; up to 30% of midwives could not accurately identify the differing educational standards, credentialing, and scope of practice for the different types of midwives licensed in the United States [30]. State variation in scope of practice may also contribute to inaccuracy. A 2020 survey of midwives found only 75% of midwives in clinical practice had hospital privileges, and only 37.5% had full medical staff privileges [31]. Without full medical staff privileges, midwives rely on physician colleagues to admit their patients and the care may then be attributed to the physician for billing purposes. This likely contributes to the underreporting of midwife-attended births on birth certificates [32].

This study was limited to the data available. This study could not verify the certification and education of providers who did not include information in the credential field. Though the AMCB data provides the most accurate count of all midwives, it does not necessary reflect the distribution of midwives currently in clinical practice. Similarly, each provider receives a single NPI and carries it forward through their career. Though changes should be reported within 30 days, there was not data available to verify the location data was up to date in the NPI data file [33]. Variation in state licensure of midwives may mean some midwives remained misclassified.

### Public health implications

This study demonstrated a method that can be used to more accurately quantify the size and distribution of the midwifery workforce. The NPI data includes practice location, allowing this method of quantifying the midwifery workforce to be used in the measurement of health provider shortage areas, including shortages of maternity care providers. This method may help direct federal funding to expand the midwifery workforce through educational grants and funding for federally qualified health centers.

### Conclusions for practice

This study demonstrated that it is possible to recode the National Provider Identifier Dataset to obtain a more accurate count of the midwifery workforce. Researchers can use the method presented in this study to improve the accuracy of evidence about the midwifery workforce.

### Supplementary Information

**Additional file 1.** **Supplement.** SAS Program to recode the NPI Datafile to identify advanced practice midwives misclassified as midwives, lay midwives, or nurse practitioners.

## Data Availability

The datasets analyzed during the current study are available from The Centers for Medicare & Medicaid at https://download.cms.gov/nppes/NPI_Files.html.
