# Supplementary material for: Evaluation of a method to identify midwives in national provider identifier data
Source: BMC Pregnancy Childbirth. 2023 Nov 22;23:809. doi: 10.1186/s12884-023-06122-2 (PMC10664267; doi:10.1186/s12884-023-06122-2)
Supplement: Supplementary file 1 — Additional file 1. Supplement. SAS Program to recode the NPI Datafile to identify advanced practice midwives misclassified as midwives, lay midwives, or nurse practitioners. [file 12884_2023_6122_MOESM1_ESM.pdf]

Supplement: SAS Program to recode the NPI Datafile to identify advanced practice midwives misclassified as midwives, lay midwives, or nurse practitioners.

```
*****************************************************;
* This program will recode to find advanced practice
* midwives by searching credential text of all three
* midwife taxonomy codes and the nurse practitioner
* taxonomy code;
* Advanced practice midwives are identified by
* credentials that indicate certification with
* the American Midwifery Certification Board (AMC)
* or training *consistent with nurse-midwifery;
* After running this program, Advanced practice midwives
* can be identified if AdvancedMidwife=1;
* This code was created with the NPI download for
* November, 2021;
* This program was validated with the NPI
* download for August, 2022 and compared to the
* the AMCB Report from August 2022;
* Program author [Blinded for Review],
* [Contact information Blinded for Review];
*****************************************************;

data NPI.recode1;
Set NPI.NPI_FULL;
IF Healthcare_Provider_Taxonomy_Co='176B00000X' or
Healthcare_Provider_Taxonomy_Co='367A00000X' or
Healthcare_Provider_Taxonomy_Co='175M00000X' or
Healthcare_Provider_Taxonomy_Co='363L00000X';
CNM=0;
CM=0;
CPM=0;
Midwife=0;
Advancedmidwife=0;


*Step one: look at NP and PA Files;
IF Healthcare_Provider_Taxonomy_Co='363L00000X' Then DO;
If find(Provider_Credential_Text, 'CNM') or
find(Provider_Credential_Text, 'C.N.M') or
find(Provider_Credential_Text, 'C.N,M') or
find(Provider_Credential_Text, 'CMN')or
find(Provider_Credential_Text, 'NMW') or
find(Provider_Credential_Text, 'N.M.W')
Then CNM = 1;

if find(Provider_Credential_Text, 'CM') or
find(Provider_Credential_Text, 'C.M')
Then CM = 1;
END;

*Step two: Identify Midwives by Credential;
*CNMs and CMs for Advanced midwife categories;

IF Healthcare_Provider_Taxonomy_Co='367A00000X' or
Healthcare_Provider_Taxonomy_Co='176B00000X' or
Healthcare_Provider_Taxonomy_Co='175M00000X'
```

```
Then Do;

If find(Provider_Credential_Text, 'CNM') or
find(Provider_Credential_Text, 'C.N.M') or
find(Provider_Credential_Text, 'C.N,M') or
find(Provider_Credential_Text, 'C .N.M') or
find(Provider_Credential_Text, 'C,N,M') or
find(Provider_Credential_Text, 'C.NM') or
find(Provider_Credential_Text, 'C.N. M') or
find(Provider_Credential_Text, 'CMN') or
find(Provider_Credential_Text, 'NMW') or
find(Provider_Credential_Text, 'N.M.W') or
find(Provider_Credential_Text, 'LNM') or
find(Provider_Credential_Text, 'L.N.M') or
find(Provider_Credential_Text, 'RN') or
find(Provider_Credential_Text, 'R.N') or
find(Provider_Credential_Text, 'NU') or
find(Provider_Credential_Text, 'BSM') or
find(Provider_Credential_Text, 'BSN') or
find(Provider_Credential_Text, 'FNP') or
find(Provider_Credential_Text, 'NP') or
find(Provider_Credential_Text, 'N.P') or
find(Provider_Credential_Text, 'DNP') or
find(Provider_Credential_Text, 'MSN') or
find(Provider_Credential_Text, 'ADVANCED') or
find(Provider_Credential_Text, 'APR') or
find(Provider_Credential_Text, 'APN') or
find(Provider_Credential_Text, 'WHNP') or
find(Provider_Credential_Text, 'W.H.N.P.') or
find(Provider_Credential_Text, 'A.P.')
Then CNM = 1;

*Recode for CMs;
if find(Provider_Credential_Text, 'CM') or
find(Provider_Credential_Text, 'C.M')
Then CM = 1;

*Recode for CPM, DEM, Traditional Midwife for Midwife Category;
if find(Provider_Credential_Text, 'CPM') or
find(Provider_Credential_Text, 'C.P.M') or
find(Provider_Credential_Text, 'CMP') or
find(Provider_Credential_Text, 'DEM') or
find(Provider_Credential_Text, 'CDM') or
find(Provider_Credential_Text, 'TM') or
find(Provider_Credential_Text, 'DIRECT') or
find(Provider_Credential_Text, 'D.E.M') or
find(Provider_Credential_Text, 'LM') or
find(Provider_Credential_Text, 'LICENSED') or
find(Provider_Credential_Text, 'LICENCED') or
find(Provider_Credential_Text, 'LIC') or
find(Provider_Credential_Text, 'L. M') or
find(Provider_Credential_Text, 'L.M') or
find(Provider_Credential_Text, 'LDM') or
find(Provider_Credential_Text, 'L.D.M') or
find(Provider_Credential_Text, 'LAY') or
find(Provider_Credential_Text, 'NHCM') or
find(Provider_Credential_Text, 'TRADITIO')or
```

```
find(Provider_Credential_Text, 'NHCM') or
find(Provider_Credential_Text, 'NH-CM') or
find(Provider_Credential_Text, 'RM') or
find(Provider_Credential_Text, 'NH')
Then CPM = 1;
End;

*Adjust for NH language;

IF Provider_License_Number_State_C='NH' Then Do;
if find(Provider_Credential_Text, 'CM') or
find(Provider_Credential_Text, 'NHCM') or
find(Provider_Credential_Text, 'NH-CM') or
find(Provider_Credential_Text, 'NH') or
find(Provider_Credential_Text, 'C.M')
Then CM = 0 AND CPM=1;
END;


*Begin recode based on certification;

IF CNM=1 or CM=1 THEN AdvancedMidwife=1;
IF CPM=1 Then Midwife=1;

*Recode for states that only recognize Advanced Practice Midwives;
If Healthcare_Provider_Taxonomy_Co='175M00000X' or
Healthcare_Provider_Taxonomy_Co='176B00000X' Then Do;
If
Provider_License_Number_State_C='NY' OR
Provider_License_Number_State_C='CT' OR
Provider_License_Number_State_C='GA' OR
Provider_License_Number_State_C='IL' OR
Provider_License_Number_State_C='IA' OR
Provider_License_Number_State_C='KS' OR
Provider_License_Number_State_C='MS' OR
Provider_License_Number_State_C='IA' OR
Provider_License_Number_State_C='MO' OR
Provider_License_Number_State_C='NE' OR
Provider_License_Number_State_C='NV' OR
Provider_License_Number_State_C='NC' OR
Provider_License_Number_State_C='ND' OR
Provider_License_Number_State_C='OH' OR
Provider_License_Number_State_C='NC'
THEN AdvancedMidwife=1;
END;

*Identify records without credential indicated to remain as categorized;
If Healthcare_Provider_Taxonomy_Co='367A00000X' and
(CPM=0 AND CNM=0 AND CM=0) THEN AdvancedMidwife=1;

IF (Healthcare_Provider_Taxonomy_Co='176B00000X' or
Healthcare_Provider_Taxonomy_Co='175M00000X') and
(CPM=0 AND CNM=0 AND CM=0 AND AdvancedMidwife=0) THEN Midwife=1;

*Create a categorical variable to count all midwives
Some midwives have more than one credential
This categorical variable will provide a count
```

```
of all midwives with each counted only once
to prevent overcount of midwives;

Category=0;
IF AdvancedMidwife=1 THEN Category=2;
Else if Midwife=1 THEN Category=1;

RUN;
```
